# 

**Published:** 1855-07

**Authors:** 


					﻿VOL. XXXII.]
[NO. 187.
THE
WESTERN JOURNAL
OF
MEDIC™ E AND SURG-ERI
NEW SERIES.
JULY, 1855.
VOL. IV.—NO. 7
EDITED BI
LUNSFORD P. YANDELL>M. D.
PROFESSOR OF PHYSIOLOGY AND PATHOLOGICAL ANATOMY, IN THE
UNIVERSITY OF LOUISVILLE.
LOUISVILLE, KY:
PRINTED AND PUBLISHED BY J. F. BRENNAN,
Cor. of Market <& First Streets
J8®*PRICE, $3 A YEAR—INVARIABLY IN ADVANCE.-POSTAGE, 2 CENTS A NUMBER.*®!
				

